# Quantification of the Antioxidant Activity of Plant Extracts: Analysis of Sensitivity and Hierarchization Based on the Method Used

**DOI:** 10.3390/antiox9010076

**Published:** 2020-01-15

**Authors:** Natividad Chaves, Antonio Santiago, Juan Carlos Alías

**Affiliations:** Department of Plant Biology, Ecology and Earth Sciences, Faculty of Science, University of Extremadura, 06006 Badajoz, Spain; asantiagk@alumnos.unex.es (A.S.); jalias@unex.es (J.C.A.)

**Keywords:** antioxidant activity methods, DPPH, FRAP, ABTS, reducing power assay, categorization of species

## Abstract

Plants have a large number of bioactive compounds with high antioxidant activity. Studies for the determination of the antioxidant activity of different plant species could contribute to revealing the value of these species as a source of new antioxidant compounds. There is a large variety of in vitro methods to quantify antioxidant activity, and it is important to select the proper method to determine which species have the highest antioxidant activity. The aim of this work was to verify whether different methods show the same sensitivity and/or capacity to discriminate the antioxidant activity of the extract of different plant species. To that end, we selected 12 species with different content of phenolic compounds. Their extracts were analyzed using the following methods: 2,2-di-phenyl-1-picrylhydrazyl (DPPH) radical scavenging capacity assay, ferric reducing (FRAP) assay, Trolox equivalent antioxidant capacity (ABTS) assay, and reducing power (RP) assay. The four methods selected could quantify the antioxidant capacity of the 12 study species, although there were differences between them. The antioxidant activity values quantified through DPPH and RP were higher than the ones obtained by ABTS and FRAP, and these values varied among species. Thus, the hierarchization or categorization of these species was different depending on the method used. Another difference established between these methods was the sensitivity obtained with each of them. A cluster revealed that RP established the largest number of groups at the shortest distance from the root. Therefore, as it showed the best discrimination of differences and/or similarities between species, RP is considered in this study as the one with the highest sensitivity among the four studied methods. On the other hand, ABTS showed the lowest sensitivity. These results show the importance of selecting the proper antioxidant activity quantification method for establishing a ranking of species based on this parameter.

## 1. Introduction

Unfavorable conditions for plants, such as extreme temperature, drought, heavy metals, nutrient deficiencies, and high salinity, generate high concentrations of reactive oxygen species (ROS), which can cause oxidative stress. To avoid this, cells have a complex antioxidant system with enzymatic and non-enzymatic elements. The molecules of the non-enzymatic system have different action mechanisms, such as enzyme inhibition, chelation of trace elements involved in the production of free radicals, reactive species uptake and activation or increase in protection through other antioxidant defenses [1]. Among these molecules, the compounds derived from secondary metabolism, specifically phenolic compounds, play a fundamental role against oxidative stress [2]. These compounds are known to act as antioxidants not only for their ability to donate hydrogen or electrons but also because they are stable radical intermediates [3]. Phenolic compounds also have protective effects on humans when the plants are consumed as food [3]. Generally, the antioxidant capacity of phenols in plant extracts is effective at low concentrations, and in humans, it is associated with the prevention of cardiovascular disease and cancer [4,5,6]. Thus, studies for the determination of the antioxidant activity of the extract of different plant species could contribute to establishing the value of these species as a source of new antioxidant compounds [7,8].

The first step to quantify the antioxidant activity of a plant extract is to select the right method [9]. There is a large variety of methods to determine this parameter [10], and the variability of experimental conditions found in the literature for each of the methods hinders such selection and the possibility of easily comparing the obtained results with those of other authors. All this makes it difficult to hierarchize plants based on the antioxidant activity of their extracts. The results of different methods for different species should be analyzed using descriptive procedures of multivariate statistical techniques to establish the best method that allows ordering or selecting the plant extracts according to their level of antioxidant activity.

The available methods to quantify antioxidant activity can be classified based on the mechanism of action by which the applied compounds stop chain-breaking reactions. They can be divided into two groups: hydrogen-atom transfer (HAT) (hydrogen atom transfer reactions) and single electron transfer (SET) (compound reduction reactions through electron transfer from an antioxidant) [11,12]. Among the SET methods, the most used are 2,2-di-phenyl-1-picrylhydrazyl (DPPH radical scavenging capacity assay), ferric reducing (FRAP) assay, Trolox equivalent antioxidant capacity (TEAC or ABTS) assay, copper reduction (CUPRAC) assay and reducing power assay (RP). Hydrogen atom transfer reaction assays include the crocin bleaching assay, the total peroxyl radical-trapping antioxidant parameter (TRAP) assay, total oxyradical scavenging capacity (TOSC) assay, and the oxygen radical absorbance capacity (ORAC) assay [11,13].

From a pool of species, selecting those with higher antioxidant activity requires knowing the method to be applied. Thus, this study aimed to determine whether different methods have the same sensitivity and/or capacity to discriminate the antioxidant activity of the extract of different species. To this end, we selected several methods with the same principle of action and which are the most used [13,14]: DPPH, FRAP, ABTS, and RP. These four SET methods were used to quantify the antioxidant activity of the methanolic extract of 12 selected plant species based on their total phenolic composition.

## 2. Materials and Methods

### 2.1. Selection of the Studied Plants

Twelve species of the Mediterranean undergrowth were selected. The Mediterranean region is characterized by heterogeneous soil and climatic conditions that have produced more than 10,000 medicinal and aromatic plant species with diverse properties worthy of further investigation [15,16]. Some of these plants have been used as folk remedies for generations after preparation in traditional ways, such as cooking, infusion, or maceration. The systematic investigation of such plants will help to define their precise pharmacological properties and to determine their value as functional foods and as a source of nutraceutical compounds, such as novel antioxidants [7,8]. This selection was based on the number of phenolic compounds they contained. On the one hand, we selected six species with high phenolic content. These species belong to the families Cistaceae (*Cistus ladanifer* L., *Cistus salvifolius* L., and *Cistus albidus* L.), Ericaceae (*Erica australis* L. and *Arbutus unedo* L.), and Anarcadiaceae (*Pistacia lentiscus* L.). The genus *Cistus* is characterized by having species with a high content of secondary metabolites [17]. Specifically, the leaves of these three species have a high content of phenolic compounds [18,19,20,21]. *Erica australis* and *Arbutus unedo* are also characterized for their high content of phenolic compounds and, particularly, condensed tannins [22,23]. Lastly, *Pistacea lentiscus* has a high content of total phenols, flavonoids, anthocyanins [24], and tannins [24,25,26].

On the other hand, we selected six species with lower phenol concentrations, which belong to the families Lamiaceae (*Teucrium fruticans* L., *Rosmarinus officinalis* L., and *Lavandula stoechas* L.), Thymelaeaceae (*Daphne gnidium* L.), Asparagaceae (*Ruscus aculeatus* L.), and Oleaceae (*Phyllirrea angustifolia* L.). The Lamiaceae are an important family of medicinal plants [27], where most species are aromatic and have essential oils [28,29]. Specifically, the three species selected for this study are rich in essential oils composed mainly of monoterpenes [20,30,31,32,33]. *Daphne gnidium* species was selected for its low, although major, concentration of flavonoids [34]. *Ruscus aculeatus* and *Phyllirrea angustifolia* species are characterized by their very low content of total phenols [20,35].

### 2.2. Plant Material Sampling Location

The location selected for the collection of the different species was in the foothills of the San Pedro mountain range, Badajoz, Spain (39°09′04″ N, 6°52′10″ W).

In April 2019, we collected 500 g of each of the species from above-ground plant material of different, randomly selected individuals. The samples were taken to the laboratory for preparation and analysis.

### 2.3. Extract Preparation

The leaves were separated from the rest of the plant material and were left to dry at room temperature. Once dry, the leaves were ground in a mechanic grinder to obtain a homogenous powder.

For the extraction, 20 g of each of the samples were weighed out, and 200 mL of methanol was added to each of these 20-g samples. They were left to macerate for 24 h in a shaker at room temperature. Then, the samples were filtered with grade 1 Whatman paper (Whatman International Ltd., Maidstone, England). The methanol was removed by evaporation at room temperature in a fume hood. The resulting extracts were stored for later analysis.

### 2.4. Total Phenols

The total phenol content was calculated using the Folin–Ciocalteu reagent assay (Merck KGaA, Darmstadt, Germany), following the method described by [36]. Aliquots of 1 mL of each extract diluted in methanol (1 mg/mL, 3 replicates per sample) had 500 µL of Folin–Ciocalteu and 6 mL of distilled water added to them. The mix was agitated for 5 min, and then 1.5 mL of Na_2_CO_3_ (20%) and 1.9 mL of distilled water were added while shaking to homogenize the dilution.

After incubation in the dark for 2 h, the absorbance was measured at 760 nm in a UV-30 spectrophotometer (GIORGIO-BORMAC SRL, Carpi, Italy). The blank was prepared by substituting the same amount of diluted extract with methanol.

The results were expressed in milligrams equivalents of gallic acid per milligram of dry weight (dw) and milligram equivalents of quercetin per milligram of dry weight. The calibration lines were established using 0.001, 0.005, 0.01, and 0.02 mg/mL of gallic acid and quercetin, respectively.

### 2.5. Determination of Antioxidant Activity Using the 2,2-Diphenyl-1-picrylhydrazyl (DPPH) Radical Scavenging Method

The antioxidant activity of the plant extracts against DPPH was determined using the method proposed by [37]. A methanolic dilution of DPPH 1 × 10^−4^ M was prepared. Aliquots of 1 mL of each sample in the methanolic extract were collected (at 4 different concentrations: 0.1, 0.5, 1, and 2 mg/mL; two replicates per sample and concentration) and had 2 mL of methanolic dilution of DPPH added.

The mix was kept in the dark at room temperature for 16 min, and absorbance was measured at 517 nm in a UV-30 spectrophotometer (GIORGIO-BORMAC SRL, Carpi, Italy). The blank was prepared with the methanolic dilution of DPPH.

The results were expressed in milligram equivalents of quercetin per milligram of dry weight. The calibration line was established using the following concentrations of quercetin: 0.001, 0.002, 0.005, 0.01, 0.02, and 0.04 mg/mL.

### 2.6. Determination of Antioxidant Activity Using the Ferric Reducing/Antioxidant Power (FRAP) Method

The FRAP assay was conducted following the method described by [38]. Aliquots of 0.2 mL of methanolic extract (at four different concentrations: 0.1, 0.5, 1, and 2 mg/mL; two replicates per sample and concentration) had 3.8 mL of FRAP reagent added. This reagent was previously prepared by mixing 10 parts of 300 mM sodium acetate buffer solution at pH 3.6, 1 part of 10 mM TPZT, and 1 part of 20 mM FeCl_3_ hexahydrate (Alfa Aesar, Kandel, Germany).

The resulting mix was incubated for 30 min at 37 °C. The absorbance increase was measured at 593 nm in a UV-30 spectrophotometer (GIORGIO-BORMAC SRL, Carpi, Italy). The blank was prepared by substituting the same amount of diluted extract with methanol.

The results were expressed in milligram equivalents of FeSO_4_ per milligram of dry weight. The calibration line was established using the following concentrations of FeSO_4_: 0.0025, 0.005, 0.01, and 0.02 mg/mL.

### 2.7. Determination of Antioxidant Activity Using the ABTS Free Radical Scavenging Method

The antioxidant activity of the study plant extracts against ABTS was determined by the method described by [39]. Radical ABTS^•+^ was prepared through oxidation of ABTS by potassium persulfate. A mixture (1:1; *v*/*v*) of ABTS (7 mM) and potassium persulfate (4.95 mM) was prepared and kept in the dark for 16 h at room temperature.

Then, the mixture was diluted with methanol until it reached absorbance values of 1–1.5 at 734 nm. Aliquots of 0.1 mL of methanolic extract of each sample (at 4 different concentrations: 0.1, 0.5, 1, and 2 mg/mL; two replicates per sample and concentration) had 3.9 mL of the ABTS^•+^ dilution added. The absorbance decrease was measured at 734 nm in a UV-30 spectrophotometer. The blank was prepared with ABTS^•+^.

The results were expressed in milligram equivalents of quercetin per milligram of dry weight. The calibration line was established using the following concentrations of quercetin: 0.00062, 0.00125, 0.0025, 0.005, 0.01, and 0.032 mg/mL.

### 2.8. Determination of Antioxidant Activity Using the Reducing Power (RP) Method

The reducing power of the plant extracts was determined by the method described by [40]. Aliquots of 1 mL of methanolic extract of each sample (at 4 different concentrations: 0.1, 0.5, 1, and 2 mg/mL; two replicates per sample and concentration) were mixed with 2.5 mL of 0.2 mM phosphate buffer solution at pH 6.6 and 2.5 mL of 1% potassium ferrocyanide.

The mixture was incubated for 20 min at 50 °C in a water bath. Then, 2.5 mL of 10% trichloroacetic acid was added, and the mixture was centrifuged a 3000 rpm for 10 min. After centrifugation, 2.5 mL of the supernatant was mixed with 2.5 mL of distilled water and 0.5 mL of 0.1% FeCl_3_.

Absorbance was measured at 700 nm in a UV-30 spectrophotometer. The blank was prepared by substituting the same amount of diluted extract with methanol. 

The results were expressed in milligram equivalents of quercetin per milligram of dry weight. The calibration line was established using the following concentrations of quercetin: 0.002, 0.0041, 0.0076, and 0.012 mg/mL.

### 2.9. Statistical Analysis

All the methods were carried out in duplicate, except for the total phenols, which were conducted in triplicate. The results were expressed as the mean of the values obtained for the replications. The dendrograms were performed with the nearest neighbor algorithm using the IBM SPSS Statistics V25.0 software. The correlation coefficients were calculated with Pearson’s test using the R statistical software. Statistical significance was established at *p* < 0.05.

## 3. Results

### 3.1. Total Phenol Content and Antioxidant Activity Quantification by Different Methods

Table 1 shows the values of the total phenol content and antioxidant activity quantified by the DPPH, ABTS, FRAP, and RP methods for the 12 plant species selected. The antioxidant activity values quantified for each of the species correspond to an extract concentration of 0.1 mg/mL. This concentration was selected for being the only one, among all the concentrations analyzed, that remained in the absorbance values of the patterns for all four methods. Appendix A shows the tables with the antioxidant activity data, quantified by the different methods, at the different extract (0.1, 0.5, 1, and 2 mg/mL) (Table A1, Table A2, Table A3 and Table A4). To facilitate the comparison between the methods, quercetin was used as a pattern, except for FRAP, which was expressed in FeSO_4_ equivalents. Total phenols were expressed in gallic acid equivalents and quercetin equivalents (GAE and QE, respectively).

As can be observed, the amounts of total phenols varied between species from 0.0364 to 0.0028 GAE (mg/mg dw) and from 0.026 to 0.002 QE (mg/mg dw). The species with the highest total phenol value was *C. salvifolius*, followed by *A. unedo* and *P. lentiscus*. On the other hand, the species with the lowest total phenol content was *R. aculeatus*, followed by *L. stoechas*, *D. gnidium,* and *T. fruticans*.

With respect to the antioxidant activity quantified by DPPH in each of the study species, the lowest value was obtained for *R. aculeatus* (0.007 QE mg/mg dw), followed by *L. stoechas* (0.008 QE mg/mg dw). The species with the highest values were *A. unedo*, *C. salvifolius*, *C. ladanifer* and *P. lenticus*, with 0.035, 0.032, 0.030 and 0.030 QE mg/mg dw, respectively. This method established a difference of around five times more antioxidant activity for *A. unedo* with respect to *R. aculeatus*. 

Regarding the antioxidant activity quantified by ABTS, the species with the highest values were *E. australis, C. salvifolius, A. unedo,* and *P. lenticus*, with 0.014, 0.013, 0.012, and 0.012 QE mg/mg dw, respectively, showing 35 times more antioxidant activity that the species with the lowest activity: *R. aculeatus* (0.0004 QE mg/mg dw). The other two species with the lowest antioxidant activity were *L. stoechas* and *T. fruticans* (0.001 QE mg/mg dw for both). With this method, a greater difference between *R. acuelatus* and *L. stoechas* was quantified.

The values obtained with RP show again *R. aculeatus* as the species with the lowest antioxidant activity (0.006 QE mg/mg dw), followed by *L. stoecha* and *D. gnidium*, with 0.008 and 0.009 QE mg/mg dw, respectively. The species with the highest antioxidant activity quantified by this method was *P. lentiscus*, with 0.031 QE mg/mg dw, establishing a 5-fold difference with *R. aculeatus*. 

Lastly, the values obtained by FRAP show that the lowest antioxidant activity was that of *R. aculeatus* (0.004 FeSO_4_ E mg/mg dw), and that the highest antioxidant activity corresponded to *C. ladanifer* and *P. lentiscus*, both with 0.071 FeSO_4_ E mg/mg dw. With this method, a 17-fold difference was established between *R. aculeatus* and *C. ladanifer* - *P. lentiscus*. 

As can be observed (Table 2), each method categorized or ordered the study species in a different way. Except for *R. aculeatus*, which was the species categorized by all the methods with the lowest antioxidant activity, the order established for the rest of the species depended on the method used. For example, the species with the highest antioxidant activity was *A. unedo*, *C. ladanifer*, *E. australis,* and *P. lentiscus,* according to DPPH, FRAP, ABTS, and RP, respectively. Likewise, excluding *R. acuelatus*, the species with the lowest activity were *L. stoechas*, *D. gnidium*, *T. fruticans,* and *L. stoechas*, according to DPPH, FRAP, ABTS, and RP, respectively. It is worth highlighting that, with FRAP, *L. stoechas* would not be categorized within the species with low antioxidant activity.

### 3.2. Correlation between Antioxidant Activity Measuring Methods

The correlation between the methods used to quantify the antioxidant activity of the 12 study species was analyzed (Table 3). A significant correlation was obtained between the different methods. It is worth highlighting that FRAP was the method with the lowest significance levels when correlated with the other methods. 

### 3.3. Cluster Analysis

**Figure 1:**
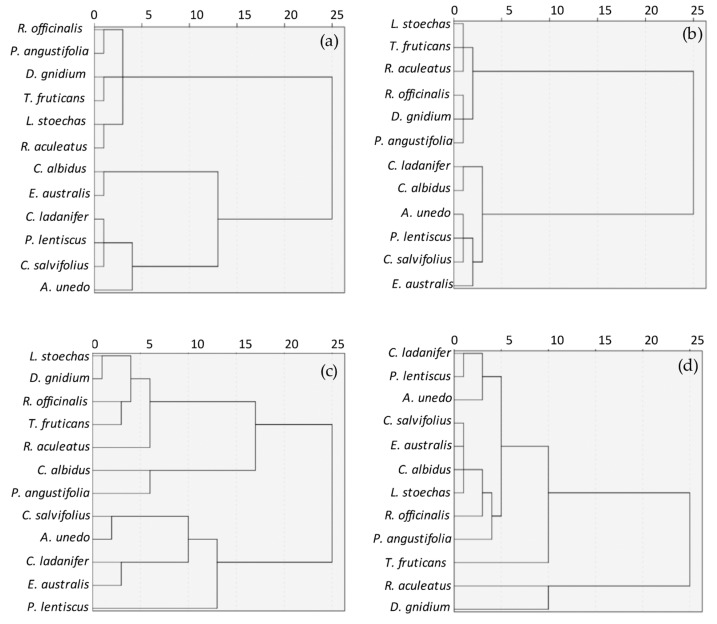
Grouping of species based on their antioxidant activity by the different methods used. (**a**) 2,2-di-phenyl-1-picrylhydrazyl (DPPH) radical scavenging capacity assay; (**b**) Trolox equivalent antioxidant capacity (ABTS) assay; (**c**) reducing power (RP) assay; (**d**) ferric reducing (FRAP) assay.

To obtain a grouping of species based on the antioxidant activity quantified by the different methods used, the values were subjected to cluster analysis. Such analysis produced the dendrograms or diagrams shown in Figure 1.

At a distance of 15 units, the dendrograms show that, for DPPH and ABTS, the species were divided into two groups, with the same species constituting these groups for both of the methods. The first group was formed by *A. unedo*, *C. salvifolius*, *P. lentiscus*, *C. ladanifer*, *C. albidus,* and *E. australis*, with values between 0.0347 and 0.0224 QE (mg/mg dw) for the DPPH method and between 0.014 and 0.009 QE (mg/mg dw) for the ABTS method. The second group comprised *R. officinalis*, *P. angustifolia*, *D. gnidium*, *T. fruticans*, *L. stoechas,* and *R. aculeatus*, with values of 0.0135–0.0068 QE (mg/mg dw) and 0.00304–0.00037 QE (mg/mg dw) for DPPH and ABTS, respectively.

The dendrogram obtained by RP divided the species into three groups. The first group was constituted by *P. lentiscus*, *C. salvifolius*, *A. unedo*, *E. australis* y *C. ladanifer*, with values between 0.0306 and 0.0220 QE mg/mg dw, the second group was formed by *C. albidus* and *P. angustifolia*, with 0.0177 and 0.0157 QE mg/mg dw, respectively, and the third group comprised *T. fruticans*, *R. officinalis*, *D. gnidium*, *L. stoechas*, and *R. aculeatus*, with values between 0.0121 and 0.0061 QE mg/mg dw.

The FRAP method established two groups of species. The first group was formed by *C. ladanifer*, *P. lentiscus*, *A. unedo*, *E. australis*, *C. salvifolius*, *C. albidus*, *L. stoechas*, *R. officinalis*, *P. angustifolia*, and *T. fruticans*, with values between 0.0713 and 0.0312 FeSO_4_ E mg/mg dw, and the second group comprised *D. gnidium* and *R. aculeatus*, with 0.0142 and 0.0038 FeSO_4_ E mg/mg dw, respectively.

## 4. Discussion

Due to the use of numerous plant species as a source of phytotherapeutic products, the study of their antioxidant activity has boomed in recent years [41,42,43]. In plants, the main compounds with antioxidant activity are phenols, as they have an aromatic ring that allows the stabilization and relocation of the unpaired electrons of their structure, thus facilitating the donation of hydrogen atoms and electrons from their hydroxyl groups [44,45]. The total phenol content varies depending on the plant species, plant tissue, developmental stage, and environmental factors, such as temperature, water stress, and light conditions [46,47].

Mediterranean plants live in habitats with extreme environmental conditions (high temperatures, water stress, and high light irradiation in summer), for which they have developed different adaptive mechanisms, both morphological and physiological. One of these mechanisms is the prevention of oxidative stress, keeping ROS under dangerous levels [48], and using them for efficient signaling [49]. Secondary metabolites of plants, specifically phenols, can adjust the concentration of ROS, thus activating a network of biochemical events to increase tolerance, hence the importance of studying the antioxidant activity of typical plant species of Mediterranean habitats.

The analysis of total phenols in the 12 Mediterranean shrub species selected shows that *C. salvifolius*, *P. lentiscus,* and *A. unedo* are the ones with the highest phenol content. These species contain approximately 12 times more phenols than *R. aculeatus*. This difference was similarly quantified by [20], who reported that the species with the highest total phenol content was *P. lentiscus*, with a value 11.6 times higher than that of the species with the lowest concentration, which was *R. aculeatus*. The 1.3-fold difference reported in that study for *P. lentiscus* and *C. albidus* with respect to *P. lentiscus* was very similar to the value obtained in the present study, which was 1.35 times higher. Other studies also show that *P. lentiscus* is one of the typical species of the Mediterranean undergrowth with the highest total phenol content [50,51,52]. Therefore, these studies confirm the validity of our results and the variability found in the total phenol content among the typical plant species of the Mediterranean undergrowth.

Phenolic compounds constitute one of the major groups of compounds known to act as primary antioxidants or free radical terminators [53], which is why it is important to quantify the amount of these compounds in the selected species.

The antioxidant activity of plant extracts can be quantified by different methods of measurement; in fact, it is recommended to use at least two different methods [54], and the results of a test system can be used to establish a ranking [11]. In our study, the four methods used to quantify the antioxidant activity of the 12 selected species showed the antioxidant capacity of these. Furthermore, all the methods presented a significant correlation between them, with high values in the Pearson’s correlation coefficient. Numerous studies have also reported this correlation [20,31,55,56,57], and some authors, such as [58], propose that, given the high correlation between the DPPH and FRAP methods, the use of more than one method to quantify antioxidant activity is redundant. These prior findings might leads us to conclude that any of the methods used in this study could be used to determine and categorize the antioxidant activity of these species, obtaining results without significant differences between them. However, the present study results show that this assertion is not correct.

On the one hand, comparing the three methods in which quercetin was used as a reference pattern (DPPH, ABTS, and RP), it can be observed that the quantified antioxidant activity was not the same. The values obtained by DPPH and RP were similar, whereas those obtained by ABTS were lower; in fact, these differences depended on the species analyzed. Specifically, DPPH quantified between 1.5 and 18.4 times more antioxidant activity than ABTS depending on the plant extract evaluated, and RP quantified between 1.6 and 16.5 times higher antioxidant activity (Table 4). Reference [59], which studied woody species of arid zones of Mexico, also reported that the values of antioxidant activity quantified by DPPH were above those obtained by ABTS and that the values of both methods varied among species. In fact, only one species, *Eucaliptus camaldulensis,* provided similar values with the two methods. Likewise, in studies conducted by [60], with medicinal plants, different values were reported for the antioxidant activity quantified by ABTS and DPPH, although, in this case, the highest values were obtained by ABTS. Thus, it is important to note that antioxidant compounds can respond in a different manner to different radical or oxidant compounds. 

On the other hand, each method categorized or ordered the study species in a different manner depending on their antioxidant activity. Except for *R. aculeatus*, which was categorized by all four methods as the species with the lowest antioxidant activity, for the rest of the species, the order established depended on the method used (Results, Table 3). Studies conducted by [20] also showed that the higher or lower activity attributed to a species depended on the methods used, such as ABTS, DPPH, and FRAP, although, in disagreement with our results, these authors [20] reported that *P. lentiscus* was the one with the highest antioxidant activity quantified by DPPH and RP. This discrepancy is due to the fact that the values of the other species studied by [20] were considerably lower than the ones attributed to *P. lentiscus*.

It is necessary to highlight that this categorization may serve as a useful tool for the selection of species with higher or lower antioxidant activity. Considering this aspect, the difference in the quantified antioxidant activity between species varies depending on the method. For instance, the relationship between the species categorized with the highest antioxidant activity in each of the methods (*A. unedo, C. ladanifer, E. australis, P. lentiscus*) and one of the species with low antioxidant activity, such as *L. stoechas*, shows a difference that varies with the method used (Table 5). Thus, with ABTS, *L. stoechas* could be discarded compared to the other species; however, with FRAP, this species should be taken into account. Consequently, extracts showing poor antioxidant properties with one concrete method should not be discarded as poor sources of antioxidants without having been tested with other methods.

Another aspect that differs among the methods used in this study is the sensitivity obtained with each of them. Using a dendrogram, we can group the species according to their antioxidant activity and then evaluate which method is the most sensitive or which of them establishes more groups or differences between the study species. From the obtained results, RP was the method that generated the largest number of groups, comparing all the methods, at a distance of 15 units. The branches of the dendrogram allow visualizing the relationships between the different species, and it was this method, RP, that established the largest number of groups at the lowest root distance. Therefore, since it better differentiated the differences and/or similarities between species, it was considered as the most sensitive method. Moreover, unlike in the other methods, this sensitivity remained constant throughout the entire range of antioxidant activity attributed to the analyzed plant species. On the other hand, ABTS was the method with the lowest sensitivity, since the dendrogram differentiated only two groups and established very little difference between the species that constituted those groups.

## 5. Conclusions

Given the results obtained in this study, it can be concluded that the four methods used can quantify the antioxidant activity of the 12 selected plant species, although the categorization established among the species depends on the method used. The methods also differ in sensitivity when establishing differences in the antioxidant activity of the species. Thus, with some of these methods, the differentiation between species is lower than that obtained with other methods. With the analyzed species, the method considered to be most sensitive, or the one that established more differences between species, was RP. These results show the importance of selecting the right method to quantify the antioxidant activity of plant extracts, especially when selecting among a group of potential species.

## Figures and Tables

**Table 1:** Phenolic content (TPC) and antioxidant activity by 2,2-di-phenyl-1-picrylhydrazyl (DPPH) radical scavenging capacity assay, ferric reducing (FRAP) assay, Trolox equivalent antioxidant capacity (ABTS) assay, and reducing power (RP) assay methods in methanolic extract of 12 selected species. Values are the mean of three replicates (TPC) and two replicates (DPPH, ABTS, RP, and FRAP) ± standard deviation.

Species	TPC	TPC	DPPH	ABTS	RP	FRAP
	(AGE mg/mg dw)	(QE mg/mg dw)				(FeSO_4_ E mg/mg dw)
*C. salvifolius*	0.036 ± 0.004	0.026 ± 0.003	0.032 ± 0.001	0.013 ± 0.0002	0.027 ± 0.001	0.057 ± 0.003
*P. lentiscus*	0.034 ± 0.003	0.024 ± 0.004	0.030 ± 0.004	0.012 ± 0.0005	0.031 ± 0.003	0.071 ± 0.002
*A. unedo*	0.032 ± 0.003	0.023 ± 0.004	0.035 ± 0.00008	0.012 ± 0.001	0.026 ± 0.002	0.065 ± 0.001
*C. ladanifer*	0.027 ± 0.003	0.019 ± 0.002	0.030 ± 0.002	0.010 ± 0.003	0.022 ± 0.003	0.071 ± 0.004
*C. albidus*	0.025 ± 0.001	0.018 ± 0.0007	0.023 ± 0.0002	0.009 ± 0.0006	0.018 ± 0.0003	0.056 ± 0.003
*E. australis*	0.025 ± 0.004	0.018 ± 0.003	0.022 ± 0.0009	0.014 ± 0.006	0.023 ± 0.001	0.058 ± 0.0008
*R. officinalis*	0.022 ± 0.001	0.015 ± 0.001	0.013 ± 0.002	0.003 ± 0.0001	0.010 ± 0.0001	0.048 ± 0.002
*P. angustifolia*	0.019 ± 0.002	0.014 ± 0.001	0.013 ± 0.0004	0.002 ± 0.001	0.016 ± 0.001	0.042 ± 0.007
*T. fruticans*	0.014 ± 0.001	0.010 ± 0.0008	0.011 ± 0.0006	0.001 ± 0.00001	0.012 ± 0.0001	0.031 ± 0.001
*D. gnidium*	0.014 ± 0.001	0.010 ± 0.0006	0.011 ± 0.0009	0.003 ± 0.0006	0.009 ± 0.0003	0.014 ± 0.0002
*L. stoechas*	0.009 ± 0.0003	0.006 ± 0.0002	0.008 ± 0.00008	0.001 ± 0.004	0.008 ± 0.0001	0.053 ± 0.01
*R. aculeatus*	0.003 ± 0.0002	0.002 ± 0.0001	0.007 ± 0.0001	0.0004 ± 0.0005	0.006 ± 0.0002	0.004 ± 0.003

AGE: gallic acid equivalents; QE: quercetin equivalents; FeSO_4_ E: FeSO_4_ equivalents; dw: dry weight.

**Table 2:** Species ordered from lower to higher antioxidant activity by method (DPPH, FRAP, ABTS, and RP). Values expressed in quercetin equivalents (mg/mg dw) (quercetin equivalents (QE)) for DPPH, ABTS and RP, and in FeSO_4_ equivalents (mg/mg dw) (FeSO_4_ E) for FRAP.

DPPH		FRAP		ABTS		PR	
*Specie*	QE	*Specie*	FeSO_4_ E	*Specie*	QE	*Specie*	QE
*R. aculeatus*	0.007	*R. aculeatus*	0.004	*R. aculeatus*	0.0004	*R. aculeatus*	0.006
*L. stoechas*	0.008	*D. gnidium*	0.014	*T. fruticans*	0.001	*L. stoechas*	0.008
*D. gnidium*	0.011	*T. fruticans*	0.031	*L. stoechas*	0.001	*D. gnidium*	0.009
*T. fruticans*	0.011	*P. angustifolia*	0.042	*P. angustifolia*	0.002	*R. officinalis*	0.010
*P. angustifolia*	0.013	*R. officinalis*	0.048	*R. officinalis*	0.003	*T. fruticans*	0.012
*R. officinalis*	0.013	*L. stoechas*	0.053	*D. gnidium*	0.003	*P. angustifolia*	0.016
*E. australis*	0.022	*C. albidus*	0.056	*C. albidus*	0.009	*C. albidus*	0.018
*C. albidus*	0.023	*C. salvifolius*	0.057	*C. ladanifer*	0.010	*C. ladanifer*	0.022
*C. ladanifer*	0.030	*E. australis*	0.058	*P. lentiscus*	0.012	*E. australis*	0.023
*P. lentiscus*	0.030	*A. unedo*	0.065	*A. unedo*	0.012	*A. unedo*	0.026
*C. salvifolius*	0.032	*P. lentiscus*	0.071	*C. salvifolius*	0.013	*C. salvifolius*	0.027
*A. unedo*	0.035	*C. ladanifer*	0.071	*E. australis*	0.014	*P. lentiscus*	0.031

**Table 3:** Correlation matrix (Pearson’s correlation coefficients) for the 12 study species.

Method	DPPH	FRAP	ABTS	RP
RP	0.944 ***	0.784 **	0.921 ***	
ABTS	0.916 ***	0.737 **		
FRAP	0.789 **			

RP: Reducing power assay; ** Significant at *p* < 0.01; *** Significant at *p* < 0.001.

**Table 4:** Relationship between antioxidant activity quantified by the DPPH and ABTS methods (DPPH/ABTS) and between the reducing power and ABTS methods (RP/ABTS).

Species	DPPH/ABTS	RP/ABTS
*C. ladanifer*	2.94	2.20
*C. salvifolius*	2.42	2.11
*C. albidus*	2.57	1.97
*R. officinalis*	4.52	3.51
*L. stoechas*	7.26	7.74
*E. australis*	1.56	1.63
*A. unedo*	2.91	2.19
*R. aculeatus*	18.4	16.5
*D. gnidium*	3.52	2.89
*T. fruticans*	10.3	11.6
*P. angustifolia*	5.73	6.67
*P. lentiscus*	2.54	2.58

**Table 5:** Relationship between the antioxidant activity of *A. unedo*, *E. australis*, *C. ladanifer*, *P. lentiscus,* and *L. stoechas* quantified by the different methods.

Method	*A. unedo* *L. stoechas*	*E.australis* *L. stoechas*	*C. ladanifer* *L. stoechas*	*P. lentiscus* *L. stoechas*
DPPH	4.51	2.91	3.81	3.92
FRAP	1.23	1.09	1.34	1.34
ABTS	11.26	13.83	11.41	11.41
RP	3.20	2.87	2.68	3.20

## Appendix A

**Table A1:** Antioxidant activity by DPPH method in methanolic extract of 12 selected species at different concentration expressed as quercetin equivalent mg/mg dw. Values are the mean of two replicates ± standard deviation.

Specie	Extract Concentration			
	0.1 mg/mL	0.5 mg/mL	1 mg/mL	2 mg/mL
*C. ladanifer*	0.030 ± 0.002	0.086 ± 0.0005	0.127 ± 0.003	0.146 ± 0.0003
*C. salvifolius*	0.032 ± 0.001	0.104 ± 0.002	0.144 ± 0.0007	−
*C. albidus*	0.023 ± 0.0002	0.072 ± 0.0001	0.111 ± 0.001	0.146 ± 0.0001
*R. officinalis*	0.013 ± 0.0004	0.044 ± 0.001	0.084 ± 0.001	0.144 ± 0.0003
*L. stoechas*	0.008 ± 0.00008	0.022 ± 0.00008	0.035 ± 0.001	0.078 ± 0.001
*E. australis*	0.022 ± 0.0009	0.078 ± 0.0005	0.121 ± 0.001	0.146 ± 0.001
*A. unedo*	0.035 ± 0.00008	0.098 ± 0.003	0.139 ± 0.0009	0.146 ± 0.00008
*R. aculeatus*	0.007 ± 0.0001	0.0094 ± 0.0001	0.013 ± 0.003	0.023 ± 0.001
*D. gnidium*	0.011 ± 0.0009	0.027 ± 0.002	0.038 ± 0.002	0.081 ± 0.002
*T. fruticans*	0.011 ± 0.0006	0.029 ± 0.001	0.052 ± 0.0001	0.094 ± 0.007
*P. angustifolia*	0.013 ± 0.004	0.034 ± 0.0005	0.065 ± 0.0006	0.115 ± 0.003
*P. lentiscus*	0.030 ± 0.002	0.107 ± 0.005	0.145 ± 0.0006	−

(−) Unquantified sample. The DPPH* radical is completely reduced to this concentration accompanied by the disappearance of the violet color.

**Table A2:** Antioxidant capacity by FRAP methods in methanolic extract of 12 selected species at different concentration expressed as FeSO_4_ equivalent mg/mg dw. Values are the mean of two replicates ± standard deviation.

Specie	Extract Concentration			
	0.1 mg/mL	0.5 mg/mL	1 mg/mL	2 mg/mL
*C. ladanifer*	0.0713 ± 0.002	0.083 ± 0.001	0.094 ± 0.002	−
*C. salvifolius*	0.0573 ± 0.003	0.079 ± 0.00007	0.096 ± 0.001	−
*C. albidus*	0.0559 ± 0.003	0.072 ± 0.002	0.078 ± 0.0005	−
*R. officinalis*	0.0481 ± 0.002	0.074 ± 0.0005	0.080 ± 0.003	0.089±0.0008
*L. stoechas*	0.0530 ± 0.01	0.071 ± 0.002	0.080 ± 0.006	0.082±0.001
*E. australis*	0.0579 ± 0.0008	0.077 ± 0.005	0.078 ± 0.00006	−
*A. unedo*	0.0653 ± 0.001	0.076 ± 0.0005	0.087 ± 0.002	−
*R. aculeatus*	0.0038 ± 0.003	0.019 ± 0.001	−	0.094±0.001
*D. gnidium*	0.0142 ± 0.0002	0.074 ± 0.005	0.089 ± 0.0002	0.117±0.003
*T. fruticans*	0.0312 ± 0.001	0.081 ± 0.002	0.093 ± 0.002	0.114±0.002
*P. angustifolia*	0.0419 ± 0.007	0.079 ± 0.0002	0.082 ± 0.0002	0.091±0.002
*P. lentiscus*	0.0711 ± 0.004	0.078 ± 0.004	−	−

(−) Unquantified sample. The absorbance is outside of the standard line at this concentration.

**Table A3:** Antioxidant capacity by ABTS methods in methanolic extract of 12 selected species at different concentration expressed as quercetin equivalent mg/mg dw. Values are the mean of two replicates ± standard deviation.

Specie	Extract Concentration			
	0.1 mg/mL	0.5 mg/mL	1 mg/mL	2 mg/mL
*C. ladanifer*	0.010 ± 0.003	−	−	−
*C. salvifolius*	0.013 ± 0.0002	−	−	−
*C. albidus*	0.009 ± 0.0006	−	−	−
*R. officinalis*	0.003 ± 0.0001	0.017 ± 0.0004	0.034 ± 0.0003	−
*L. stoechas*	0.001 ± 0.0004	0.009 ± 0.00005	0.015 ± 0.0004	−
*E. australis*	0.014 ± 0.006	−	−	−
*A. unedo*	0.012 ± 0.001	−	−	−
*R. aculeatus*	0.0004 ± 0.0005	0.002 ± 0.0008	0.005 ± 0.0002	0.010 ± 0.0007
*D. gnidium*	0.003 ± 0.0006	0.016 ± 0.0006	0.023 ± 0.0007	0.034 ± 0.00005
*T. fruticans*	0.001 ± 0.0001	0.010 ± 0.0008	0.020 ± 0.0007	0.033 ± 0.00001
*P. angustifolia*	0.002 ± 0.001	0.014 ± 0.001	0.024 ± 0.0005	−
*P. lentiscus*	0.012 ± 0.0005	−	−	−

(−) Unquantified sample. The ABTS* radical is completely reduced to this concentration accompanied by the disappearance of the green color.

**Table A4:** Antioxidant capacity by reductor power methods in methanolic extract of 12 selected species at different concentration expressed as quercetin equivalent mg/mg dw. Values are the mean of two replicates ± standard deviation.

Specie	Extract Concentration			
	0.1 mg/mL	0.5 mg/mL	1 mg/mL	2 mg/mL
*C. ladanifer*	0.022 ± 0.003	0.089 ± 0.001	0.155 ± 0.002	−
*C. salvifolius*	0.027 ± 0.001	0.125 ± 0.004	−	−
*C. albidus*	0.018 ± 0.0003	0.077 ± 0.002	0.125 ± 0.001	−
*R. officinalis*	0.010 ± 0.0001	0.052 ± 0.0005	0.101 ± 0.003	0.191 ± 0.002
*L. stoechas*	0.008 ± 0.0001	0.031 ± 0.002	0.045 ± 0.0004	0.100 ± 0.001
*E. australis*	0.023 ± 0.001	0.078 ± 0.003	0.131 ± 0.003	−
*A. unedo*	0.026 ± 0.002	0.097 ± 0.001	0.172 ± 0.001	−
*R. aculeatus*	0.006 ± 0.0002	0.011 ± 0.001	0.026 ± 0.005	0.080 ± 0.0001
*D. gnidium*	0.009 ± 0.0003	0.026 ± 0.002	0.038 ± 0.0002	0.071 ± 0.004
*T. fruticans*	0.012 ± 0.0001	0.040 ± 0.003	0.077 ± 0.001	0.144 ± 0.002
*P. angustifolia*	0.016 ± 0.001	0.052 ± 0.001	0.093 ± 0.001	0.160 ± 0.002
*P. lentiscus*	0.031 ± 0.003	0.117 ± 0.004	−	−

(−) Unquantified sample. The absorbance is outside of the standard line at this concentration.

## References

[1] Barua C.C., Sen S., Das A.S., Talukdar A., Jyoti Hazarika N., Barua A., Barua I. (2014). A comparative study of the in vitro antioxidant property of different extracts of *Acorus calamus* Linn. J. Nat. Prod. Plant Resour..

[2] Pang Y., Ahmed S., Xu Y., Beta T., Zhu Z., Shao Y., Bao J. (2018). Bound phenolic compounds and antioxidant properties of whole grain and bran of white, red and black rice. Food Chem..

[3] Nićiforović N., Mihailović V., Mašković P., Solujić S., Stojković A., Muratspahić D.P. (2010). Antioxidant activity of selected plant species; potential new sources of natural antioxidants. Food Chem. Toxicol..

[4] Duthie G.G., Duthie S.J., Kyle J.A.M. (2000). Plant polyphenols in cancer and heart disease: Implications as nutritional antioxidants. Nutr. Res. Rev..

[5] Li A.-N., Li S., Zhang Y.-J., Xu X.-R., Chen Y.-M., Li H.-B. (2014). Resources and biological activities of natural polyphenols. Nutrients.

[6] Balmus I., Ciobica A., Trifan A., Stanciu C. (2016). The implications of oxidative stress and antioxidant therapies in Inflammatory Bowel Disease: Clinical aspects and animal models. Saudi J. Gastroenterol..

[7] Miliauskas G., Venskutonis P.R., van Beek T.A. (2004). Screening of radical scavenging activity of some medicinal and aromatic plant extracts. Food Chem..

[8] Gouthamchandra K., Mahmood R., Manjunatha H. (2010). Free radical scavenging, antioxidant enzymes and wound healing activities of leaves extracts from *Clerodendrum infortunatum* L.. Environ. Toxicol. Pharmacol..

[9] Abramovič H., Grobin B., Poklar Ulrih N., Cigić B. (2017). The methodology applied in DPPH, ABTS and Folin-Ciocalteau assays has a large influence on the determined antioxidant potential. Acta Chim. Slov..

[10] Apak R., Güçlü K., Özyürek M., Karademir S.E. (2008). Mechanism of antioxidant capacity assays and the CUPRAC (cupric ion reducing antioxidant capacity) assay. Microchim. Acta.

[11] Prior R.L., Wu X., Schaich K. (2005). Standardized methods for the determination of antioxidant capacity and phenolics in foods and dietary supplements. J. Agric. Food Chem..

[12] Pérez Jiménez J. (2007). Metodología Para la Evaluación de Ingredientes Funcionales Antioxidantes: Efectos de Fibra Antioxidante de Uva en Status Antioxidante Y Parámetros de Riesgo Cardiovascular en Humanos. Ph.D. Thesis.

[13] Huang D., Ou B., Prior R.L. (2005). The chemistry behind antioxidant capacity assays. J. Agric. Food Chem..

[14] Schaich K.M., Tian X., Xie J. (2015). Hurdles and pitfalls in measuring antioxidant efficacy: A critical evaluation of ABTS, DPPH, and ORAC assays. J. Funct. Foods.

[15] Fares R., Bazzi S., Baydoun S.E., Abdel-Massih R.M. (2011). The antioxidant and antiproliferative activity of the Lebanese *Olea europaea* extract. Plant Foods Hum. Nutr..

[16] Vanzani P., Rossetto M., De Marco V., Sacchetti L.E., Paoletti M.G., Rigo A. (2011). Wild Mediterranean plants as traditional food: A valuable source of antioxidants. J. Food Sci..

[17] Papaefthimiou D., Papanikolaou A., Falara V., Givanoudi S., Kostas S., Kanellis A.K. (2014). Genus *Cistus*: A model for exploring labdane-type diterpenes’ biosynthesis and a natural source of high value products with biological, aromatic, and pharmacological properties. Front. Chem..

[18] Vogt T., Proksch P., Gülz P.-G. (1987). Epicuticular flavonoid aglycones in the genus *Cistus*, Cistaceae. J. Plant Physiol..

[19] Chaves N., Escudero J.C., Gutiérrez-Merino C. (1997). Quantitative variation of flavonoids among individuals of a *Cistus ladanifer* population. Biochem. Syst. Ecol..

[20] González-Burgos E., Gómez-Serranillos M.P. (2012). Terpene compounds in nature: A review of their potential antioxidant activity. Curr. Med. Chem..

[21] Guerreiro O., Alves S.P., Duarte M.F., Bessa R.J.B., Jerónimo E. (2015). *Cistus ladanifer* L. Shrub is rich in saturated and branched chain fatty acids and their concentration increases in the mediterranean dry season. Lipids.

[22] Peñuelas J., Castells E., Joffre R., Tognetti R. (2002). Carbon-based secondary and structural compounds in mediterranean shrubs growing near a natural CO_2_ spring. Glob. Chang. Biol..

[23] Ammar H., López S., González J.S. (2005). Assessment of the digestibility of some Mediterranean shrubs by in vitro techniques. Anim. Feed Sci. Technol..

[24] Rozin P., Spranca M., Krieger Z., Neuhaus R., Surillo D., Swerdlin A., Wood K. (2004). Preference for natural: Instrumental and ideational/moral motivations, and the contrast between foods and medicines. Appetite.

[25] Romani A., Pinelli P., Galardi C., Mulinacci N., Tattini M. (2002). Identification and quantification of galloyl derivatives, flavonoid glycosides and anthocyanins in leaves of *Pistacia lentiscus* L.. Phytochem. Anal..

[26] Lehmann J., Große-Stoltenberg A., Römer M., Oldeland J. (2015). Field spectroscopy in the vnir-swir region to discriminate between mediterranean native plants and exotic-invasive shrubs based on leaf tannin content. Remote Sens..

[27] Carović-Stanko K., Petek M., Grdiša M., Pintar J., Bedeković D., Herak Ćustić M., Satovic Z. (2016). Medicinal plants of the family Lamiaceae as functional foods—A review. Czech J. Food Sci..

[28] Lawrence B.M., Harley R.M., Reynolds T. (1992). Chemical components of Labiatae oils and their exploitation. Advances in Labiate Science.

[29] Özkan M. (2008). Glandular and eglandular hairs of *Salvia recognita* Fisch. & Mey. (*Lamiaceae*) in Turkey. Bangladesh J. Bot..

[30] Al-Sereiti M.R., Abu-Amer K.M., Sen P. (1999). Pharmacology of rosemary (*Rosmarinus officinalis* L.) and its therapeutic potentials. Indian J. Exp. Biol..

[31] Parejo I., Viladomat F., Bastida J., Rosas-Romero A., Flerlage N., Burillo J., Codina C. (2002). Comparison between the radical scavenging activity and antioxidant activity of six distilled and nondistilled mediterranean herbs and aromatic plants. J. Agric. Food Chem..

[32] Boix Y.F., Victório C.P., Defaveri A.C.A., Arruda R.D.C.D.O., Sato A., Lage C.L.S. (2011). Glandular trichomes of *Rosmarinus officinalis* L.: Anatomical and phytochemical analyses of leaf volatiles. Plant Biosyst..

[33] Djabou N., Lorenzi V., Guinoiseau E., Andreani S., Giuliani M.-C., Desjobert J.-M., Muselli A. (2013). Phytochemical composition of Corsican *Teucrium* essential oils and antibacterial activity against foodborne or toxi-infectious pathogens. Food Control.

[34] Chaabane F., Boubaker J., Loussaif A., Neffati A., Kilani-Jaziri S., Ghedira K., Chekir-Ghedira L. (2012). Antioxidant, genotoxic and antigenotoxic activities of *Daphne gnidium* leaf extracts. BMC Complement. Altern. Med..

[35] Romani A., Baldi A., Mulinacci N., Vincieri F.F., Tattini M. (1996). Extraction and identification procedures of polyphenolic compounds and carbohydrates in phillyrea (*Phillyrea angustifolia* L.) leaves. Chromatographia.

[36] Singleton V.L., Rossi J.A. (1965). Colorimetry of total phenolics with phosphomolybdic-phosphotungstic acid reagent. Am. J. Enol. Vitic..

[37] Katalinic V., Milos M., Kulisic T., Jukic M. (2006). Screening of 70 medicinal plant extracts for antioxidant capacity and total phenols. Food Chem..

[38] Benzie I.F.F., Strain J.J. (1996). The ferric reducing ability of plasma (FRAP) as a measure of “Antioxidant Power”: The FRAP assay. Anal. Biochem..

[39] Stratil P., Klejdus B., Kubáň V. (2006). Determination of total content of phenolic compounds and their antioxidant activity in vegetables evaluation of spectrophotometric methods. J. Agric. Food Chem..

[40] Oyaizu M. (1986). Studies on products of browning reaction: Antioxidative activities of products of browning reaction prepared from glucosamine. Jpn. J. Nutr..

[41] Dávalos A., Gómez-Cordovés C., Bartolomé B. (2003). Commercial Dietary Antioxidant Supplements Assayed for Their Antioxidant Activity by Different Methodologies. J. Agric. Food Chem..

[42] Moon J.-K., Shibamoto T. (2009). Antioxidant assays for plant and food components. J. Agric. Food Chem..

[43] Londoño-Londoño J. (2012). Antioxidantes: Importancia biológica y métodos para medir su actividad. Desarrollo Y Transversalidad. Serie Lasallista Investigación Y Ciencia.

[44] Rice-Evans C., Miller N., Paganga G. (1997). Antioxidant properties of phenolic compounds. Trends Plant Sci..

[45] Cömert E.D., Gökmen V. (2017). Antioxidants bound to an insoluble food matrix: Their analysis, regeneration behavior, and physiological importance. Compr. Rev. Food Sci..

[46] Upadrasta L., Mukhopadhyay M., Banerjee R., Sabu A., Roussos S., Aguilar C.N. (2011). Tannins: Chemistry, biological properties and biodegradation. Chemistry and Biotechnology of Polyphenols.

[47] Zlatić N., Jakovljević D., Stanković M. (2019). Temporal, plant part, and interpopulation variability of secondary metabolites and antioxidant activity of *Inula helenium* L.. Plants.

[48] Tardieu F., Tuberosa R. (2010). Dissection and modelling of abiotic stress tolerance in plants. Curr. Opin. Plant Biol..

[49] Hadacek F., Bachmann G., Engelmeier D., Chobot V. (2011). Hormesis and a chemical Raison D’être for secondary plant metabolites. Dose Response.

[50] Aidi Wannes W., Mhamdi B., Sriti J., Ben Jemia M., Ouchikh O., Hamdaoui G., Kchouk M., Marzouk B. (2010). Antioxidant activities of the essential oils and methanol extracts from myrtle (*Myrtus communis* var. italica L.) leaf, stem and flower. Food Chem. Toxicol..

[51] Djeridane A., Yousfi M., Nadjemi B., Boutassouna D., Stocker P., Vidal N. (2006). Antioxidant activity of some algerian medicinal plants extracts containing phenolic compounds. Food Chem..

[52] Amessis-Ouchemoukh N., Madani K., Falé P.L.V., Serralheiro M.L., Araújo M.E.M. (2014). Antioxidant capacity and phenolic contents of some Mediterranean medicinal plants and their potential role in the inhibition of cyclooxygenase-1 and acetylcholinesterase activities. Ind. Crops Prod..

[53] Fawole O.A., Ndhlala A.R., Amoo S.O., Finnie J.F., Van Staden J. (2009). Antiinflammatory and phytochemical properties of twelve medicinal plants used for treating gastrointestinal ailments in South Africa. J. Ethnopharmacol..

[54] Milan C., Hana C., Petko D., Maria K., Anton S., Antonín L. (2010). Different methods for control and comparison of the antioxidant properties of vegetables. Food Control.

[55] Thaipong K., Boonprakob U., Crosby K., Cisneros-Zevallos L., Byrne D.H. (2006). Comparison of ABTS, DPPH, FRAP, and ORAC assays for estimating antioxidant activity from guava fruit extracts. J. Food Compos. Anal..

[56] Wojdylo A., Oszmianski J., Czemerys R. (2007). Antioxidant activity and phenolic compounds in 32 selected herbs. Food Chem..

[57] Dudonné S., Vitrac X., Coutière P., Woillez M., Mérillon J.-M. (2009). Comparative study of antioxidant properties and total phenolic content of 30 plant extracts of industrial interest using DPPH, ABTS, FRAP, SOD, and ORAC assays. J. Agric. Food Chem..

[58] Garry C., Kang Nee T., Christophe W., Jeffrey F. (2013). High correlation of 2,2-diphenyl-1-picrylhydrazyl (DPPH) radical scavenging, ferric reducing activity potential and total phenolics content indicates redundancy in use of all three assays to screen for antioxidant activity of extracts of plants from the malaysian rainforest. Antioxidants.

[59] Wong-Paz J.E., Contreras-Esquivel J.C., Rodríguez-Herrera R., Carrillo-Inungaray M.L., López L.I., Nevárez-Moorillón G.V., Aguilar C.N. (2015). Total phenolic content, in vitro antioxidant activity and chemical composition of plant extracts from semiarid Mexican region. Asian Pac. J. Trop. Med..

[60] Rajurkar N.S., Hande S.M. (2011). Estimation of phytochemical content and antioxidant activity of some selected traditional indian medicinal plants. Indian J. Pharm. Sci..

